# Diabetes care in China: Innovations and implications

**DOI:** 10.1111/jdi.13908

**Published:** 2022-09-19

**Authors:** Weiping Jia

**Affiliations:** ^1^ National Office for Primary Diabetes Care, Shanghai Technical Center for Diabetes Prevention and Clinical Care, Shanghai Key Laboratory of Diabetes Mellitus, Department of Endocrinology and Metabolism, Shanghai Clinical Center for Diabetes, Shanghai Key Clinical Center for Metabolic Disease Shanghai Jiao Tong University Affiliated Sixth People's Hospital, Shanghai Diabetes Institute Shanghai China

Diabetes has become an important public health concern in China, with a rapid increase in prevalence from 0.67% in 1980 to 9.7% in 2007 and, then, a slight increase to 11.2% in 2017^1^. China currently has the largest number of people with diabetes worldwide, with nearly one‐quarter of global cases being found in China. In 2019, approximately 824,000 adults were estimated to die as a result of diabetes and related complications, with its estimated diabetes‐related health expenditures ranking second to that of the USA^1^. The increasing prevalence of diabetes, high avoidable morbidity and mortality as a result of diabetes and diabetic complications, and related substantial economic burden make diabetes one of the greatest health challenges in China.

Due to an uneven distribution of medical resources in China, with a lack of high‐quality human resources and insufficient capacity in primary healthcare, the burden of non‐communicable diseases on the whole society is high. Since 2009, the Chinese government has started the deepening of health system reform, and included type 2 diabetes as one of the two major non‐communicable diseases into basic public health services to strengthen the public health system^2^. Regarding diabetes, the basic public health services package includes screening, regular follow up including a minimum of four blood glucose tests per year and blood pressure measurement, and health education. In addition, it was promoted to expand education and training for general practitioners (GPs); create a contracting system for GPs to improve the quality of primary healthcare; and establish a tiered service delivery system including tertiary, secondary and primary healthcare providers to better use of health resources, and the integration of healthcare delivery among different levels of health institutions.

In Shanghai, the diabetes management services provided through community health service centers (CHSCs) have been started as pilot work since 2005. Since 2007, as a result of suboptimal diabetes management found in primary care, the Shanghai Jiao Tong University Affiliated Sixth People's Hospital has started a pilot model, an integrated hospital‐community hierarchical diabetes management system, through seven CHSCs and a tertiary general hospital in Shanghai^2^. In this pilot model, the tertiary general hospital provided technical training for GPs and nurses in CHSCs to improve their capability for screening, diagnosis, treatment and health education of diabetes.

Through the implementation of this model, the proportions of those achieving glycemic targets and receiving chronic complication screening have increased^2^. In addition, a multidisciplinary combined screening, diagnosis, treatment and follow‐up platform for diabetes has been developed, and it has facilitated a close collaboration between tertiary general hospitals and primary healthcare institutions. It makes it feasible for high‐risk general population and patients to receive timely and appropriate health education, diagnosis, and treatment services, and facilitates a better allocation of health service resources. With the continuous promotion of basic public health services, approximately 630,000 patients with diabetes were being managed at CHSCs by 2016^1^. However, it does not give enough attention to improve synergy between public health initiatives and medical services.

Based on the previous experience of the integrated hospital‐community hierarchical diabetes management system, the Shanghai Municipal Government initiated a large project to develop a Shanghai Integrated Diabetes Prevention and Care System, the “Shanghai Integration Model (SIM)” (Figure 1), starting from 2015^1^. The Shanghai Jiao Tong University Affiliated Sixth People's Hospital has been accredited as the Shanghai Technical Center for Diabetes Prevention and Clinical Care, with a close collaboration of the Shanghai Municipal Center for Disease Control & Prevention, and the Shanghai Eye Disease Prevention and Treatment Center. The SIM set out to promote the integration of primary and specialty care, as well as the content of diabetes care and public health services, including health education, screening, diagnosis and treatment, regular primary care, needed specialty care, care for complications, and self‐management through peer support.

**Figure 1:**
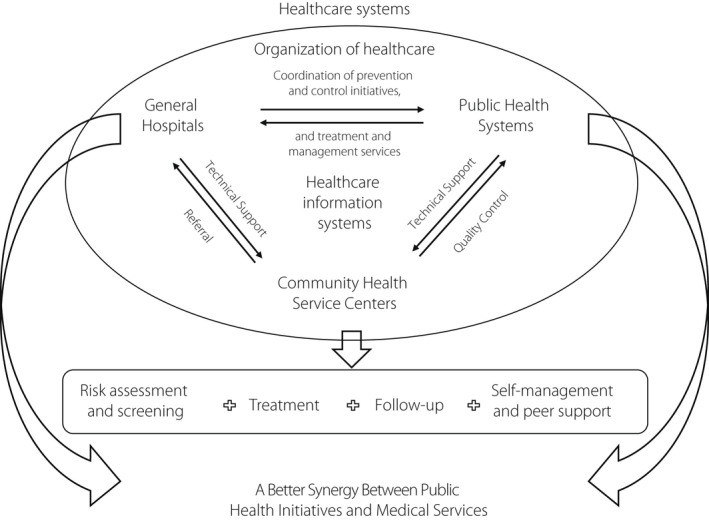
Shanghai integration model framework.

All 240 CHSCs in 16 districts have been invited to participate in this project, with funding support from the Shanghai government. Through this project, prediabetes and diabetes screening for those at high risk, and complications screening for patients with diabetes were implemented in CHSCs, facilitated by the use of city‐wide electronic patient registries, care coordination, and training and technical support from secondary and tertiary general hospitals, to enhance the capability of diabetes care in all CHSCs. In this project, approximately 325,000 individuals at high risk for diabetes have been screened for diabetes with the rate of newly‐diagnosed diabetes and prediabetes of 11.4 and 14.8%^3^. In addition, the rates of chronic diabetic complication screening have significantly increased, which was rarely implemented in primary healthcare institutions in China. A total of 173,235 patients with type 2 diabetes were screened for chronic complications in 2017, with 44.7% of patients having one or more microvascular complications^1^. For the patients found with severe complications through screening, a rapid referral to general hospitals for needed specialty care is implemented. Approximately 4,000 patients with diabetic retinopathy and 20,000 patients with diabetic kidney diseases receive referrals to secondary or tertiary general hospitals^3^.

Regarding self‐management support, the integration of care and public health roles of CHSCs in chronic care provides a useful setting for integrating peer support into regular care, while peer support facilitates the objectives of the SIM, encouraging appropriate primary as well as specialty care, helping patients to integrate medical care, healthy lifestyle and self‐management in their daily lives^4^. After 2017, the SIM was incorporated into the framework for the integrated community management of chronic diseases that guided routine diabetes management work in CHSCs in Shanghai.

With the global internet penetration, digital technologies have been increasingly adopted to improve glycemic control, especially in areas with limited health resources. A mobile health (mHealth)‐based digital platform named Road to Hierarchical Diabetes Management at Primary Care Settings in China (ROADMAP) was designed as a further extension of the SIM. It was implemented and evaluated from 2017 to 2019 in diverse primary healthcare settings, including a total of 864 communities in 144 counties across 25 provinces covering both urban and rural areas^5^. The intervention included a tiered care team‐delivered mHealth‐mediated service package, initiated by monthly blood glucose monitoring at each structured clinic visit, capacity building and quarterly performance review strategies, to improve the quality of primary healthcare for diabetes. The digital application created a link connecting both GPs from primary healthcare clinics in communities and specialty doctors from county hospitals, and built a bridge to minimize the gap in diabetes care between urban and rural areas. After 1‐year follow up, the mHealth‐enabled hierarchical diabetes management intervention effectively improved diabetes control in primary healthcare, leading to an absolute improvement in the glycated hemoglobin control rate of 7.0% and a relative improvement of 18.6%^5^. ROADMAP is the largest randomized controlled trial in testing the effectiveness and safety of an mHealth‐based diabetes management with a diverse coverage of primary care settings, and the effectiveness of this intervention shows the potential of this model to be applied in the primary healthcare system and transferred to other chronic conditions management in similar contexts in China, as well as in other low‐ and middle‐income countries, which are struggling to meet the gaps in chronic disease management, including diabetes.

In 2019, the Chinese government further promoted the Healthy China 2030 blueprint, extending the health system reforms through a variety of preventive and capacity reforms, with the integration of health promotion, disease prevention and treatment, and health management, requiring engagement from all sectors in society. In the Healthy China 2030 blueprint, the Diabetes Prevention and Treatment Initiative has been listed as one of the 15 major initiatives. The practice of the integrated hospital‐community hierarchical diabetes management system, and the SIM in Shanghai, and the ROADMAP across 25 provinces covering both urban and rural areas in China showed that with strengthened integration and collaboration of primary healthcare and specialty care, a better synergy between public health initiatives and medical services, and use of innovative intervention strategies in rural areas, care of patients with diabetes could be substantially enhanced.

## DISCLOSURE

The author declares no conflict of interest.

Approval of the research protocol: N/A.

Informed consent: N/A.

Registry and the registration no. of the study/trial: N/A.

Animal studies: N/A.
